# Supplementary material for: Dalbergia ecastaphyllum (L.) Taub. and Symphonia globulifera L.f.: The Botanical Sources of Isoflavonoids and Benzophenones in Brazilian Red Propolis
Source: Molecules. 2020 Apr 28;25(9):2060. doi: 10.3390/molecules25092060 (PMC7249054; doi:10.3390/molecules25092060)
Supplement: Supplementary file 1 [file molecules-25-02060-s001.pdf]

Dalbergia ecastaphyllum (L.) Taub. and *Symphonia globulifera* L.f.: the botanical sources of isoflavonoids and benzophenones in Brazilian red propolis

Gari Vidal Ccana-Ccapatinta [1], Jennyfer Andrea Aldana Mejía [1], Matheus Hikaru Tanimoto [1], Milton Groppo [2], Jean Carlos Andrade Sarmento de Carvalho [3] and Jairo Kenupp Bastos [1,*]

[1] Laboratory of Pharmacognosy, School of Pharmaceutical Sciences of Ribeirão Preto, University of São Paulo (USP), Av. do Café s/n, 14040-903, Ribeirão Preto, SP, Brazil

[2] Laboratory of Plant Systematics, Department of Biology, Faculty of Philosophy, Sciences and Letters at Ribeirão Preto, USP, Av. dos Bandeirantes 3900, 14040-901, Ribeirão Preto, SP, Brazil

[3] Cooperativa de Apicultores de Canavieiras (COAPER), Av. Burundanga 1900, 45860-000, Canavieiras, BA, Brasil

* Correspondence: jkbastos@fcfrp.usp.br; Tel.: +55-16-3315-4230

Graphical abstract

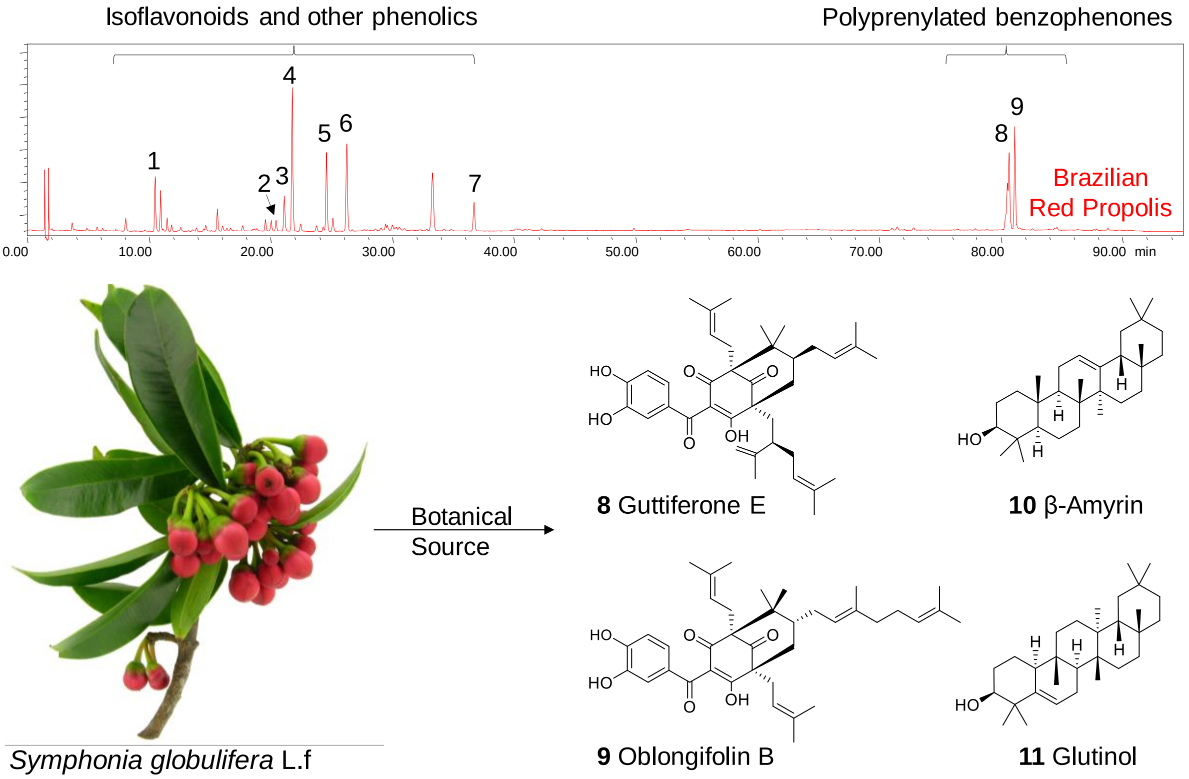

Supplementary Material:

Figure 1. ^{1}H NMR spectra of guttiferone E (500 MHz, CD_3OD + 0.1% TFA)…………..3

Figure 2. ^{13}C NMR spectra of guttiferone E (125 MHz, CD_3OD + 0.1% TFA)…………3

Figure 3. HSQC NMR spectra of guttiferone E (500 MHz, CD_3OD + 0.1% TFA)……...4

Figure 4. HMBC NMR spectra of guttiferone E (500 MHz, CD_3OD + 0.1% TFA)……..4

Figure 5. ^{1}H NMR spectra of oblongifolin B (500 MHz, CD_3OD + 0.1% TFA)…………5

Figure 6. ^{13}C NMR spectra of oblongifolin B (125 MHz, CD_3OD + 0.1% TFA)………...5

Figure 7. HSQC NMR spectra of oblongifolin B (500 MHz, CD_3OD + 0.1% TFA)…….6

Figure 8. HMBC NMR spectra of oblongifolin B (500 MHz, CD_3OD + 0.1% TFA)……6

Figure 9. Negative mode HRESIMS full scan mass spectra of guttiferone E…………7

Figure 10. Negative mode HRESIMS full scan mass spectra of oblongifolin B………7

Table 1. NMR data of compound 9 compared to literature data of oblongifolins……..8

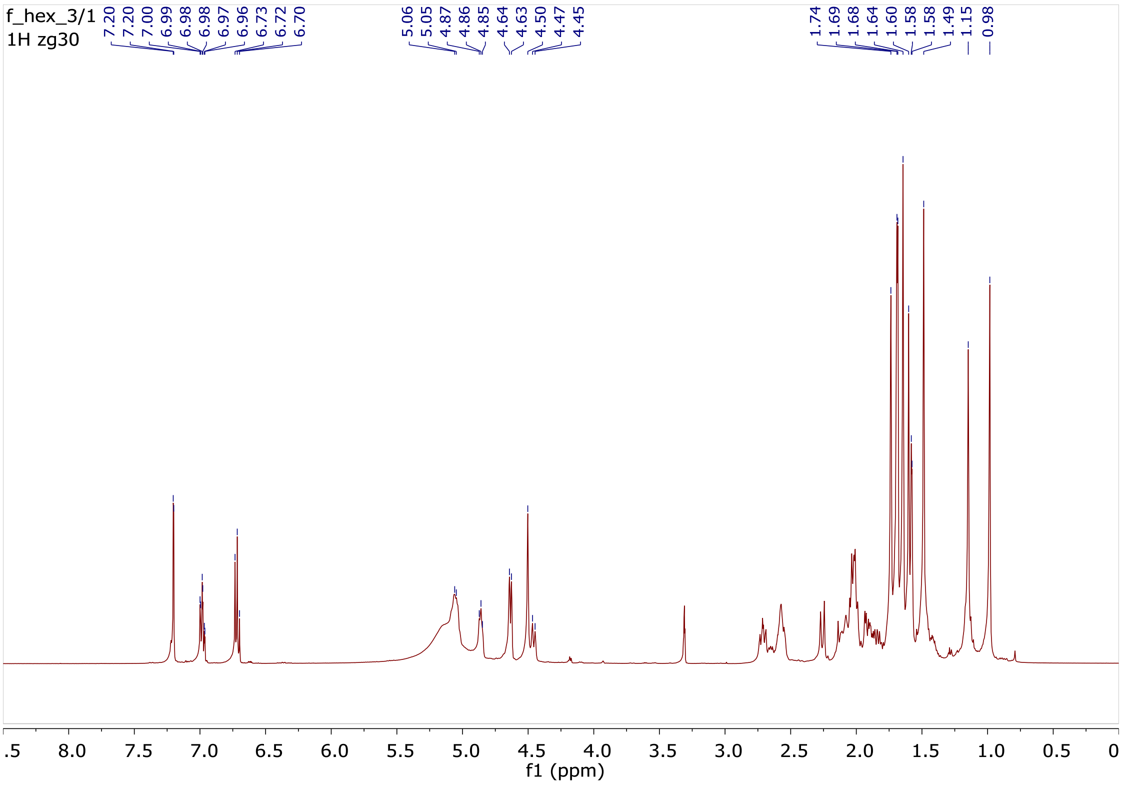

Figure 1. ^{1}H NMR spectra of guttiferone E (**8**), 500 MHz, CD_3OD + 0.1% TFA.

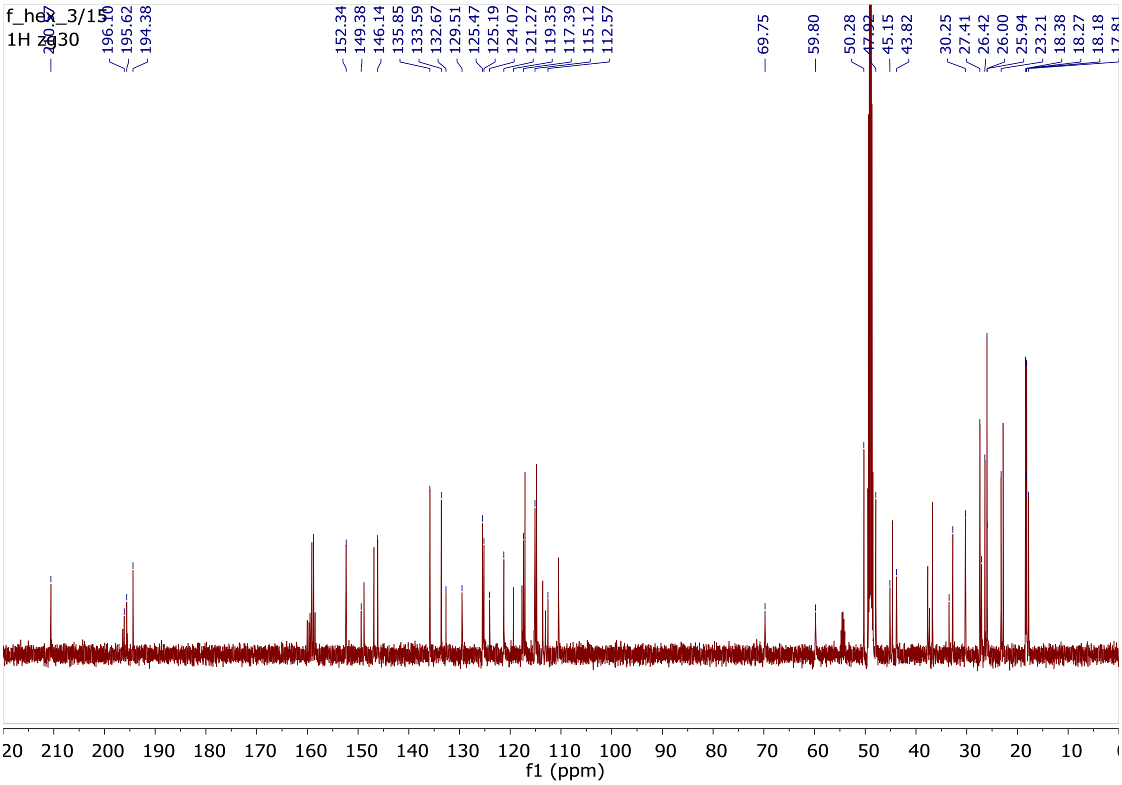

Figure 2. ^{13}C NMR spectra of guttiferone E (**8**), 125 MHz, CD_3OD + 0.1% TFA.

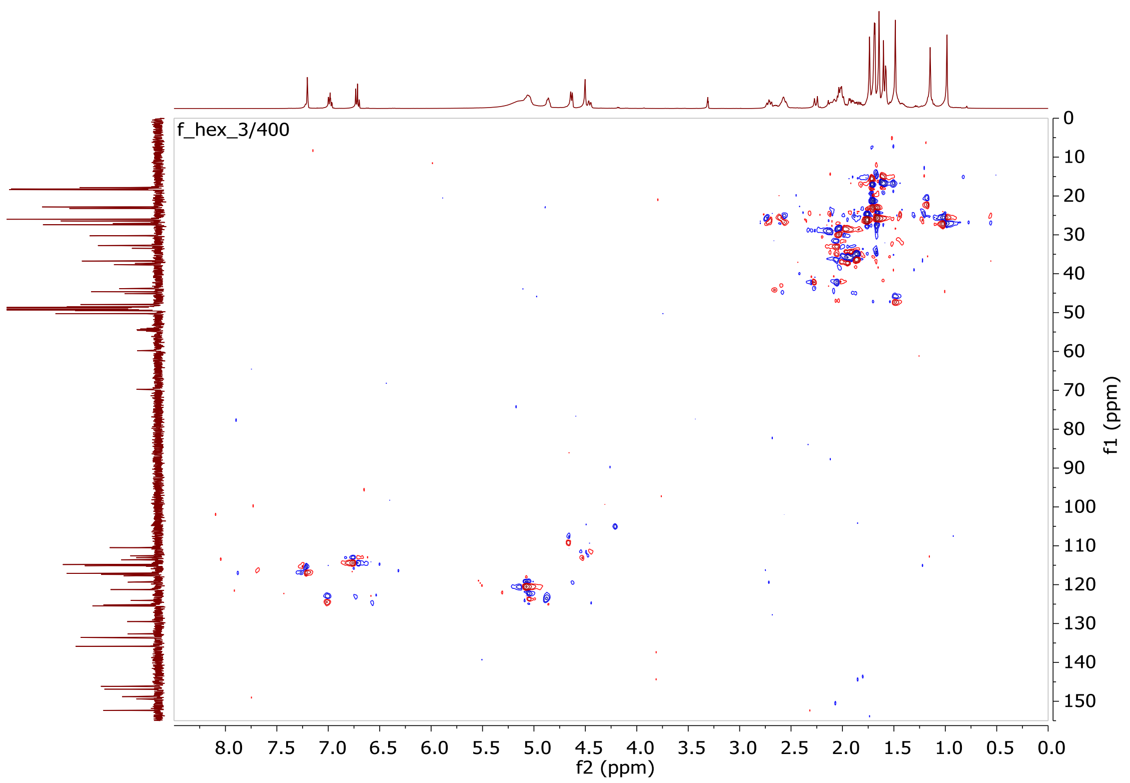

Figure 3. HSQC NMR spectra of guttiferone E (**8**), 500 MHz, CD_3OD + 0.1% TFA.

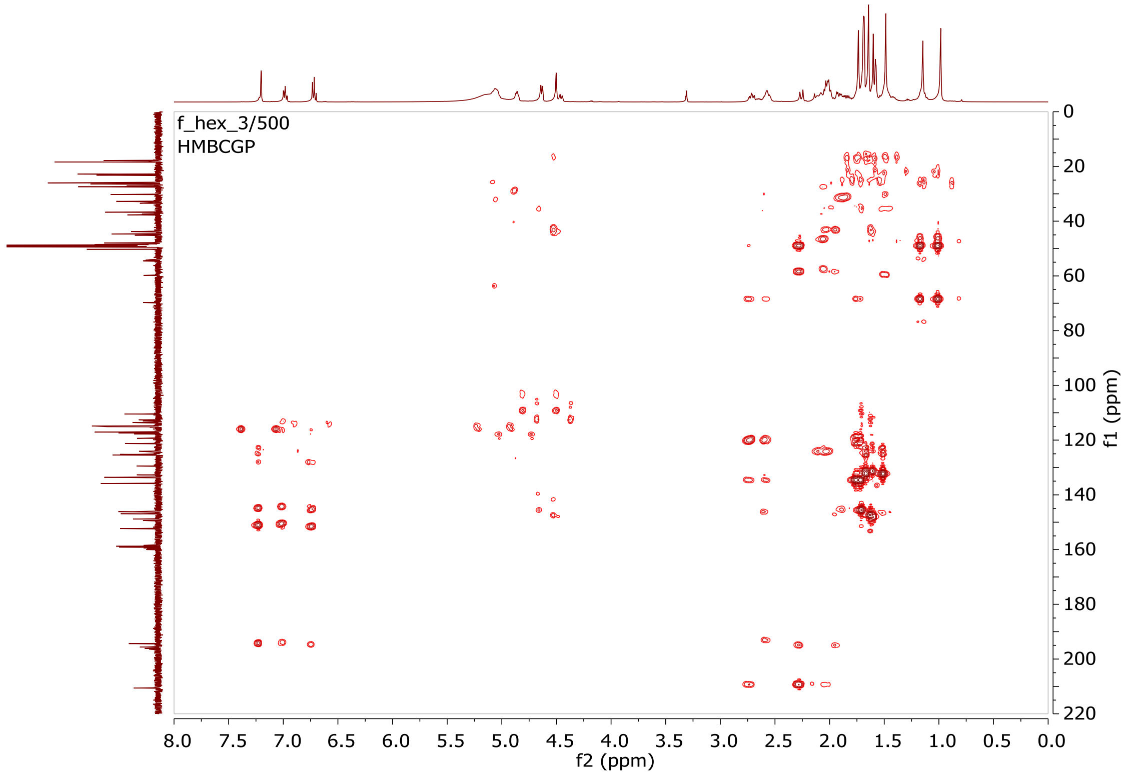

Figure 4. HMBC NMR spectra of guttiferone E (**8**), 500 MHz, CD_3OD + 0.1% TFA.

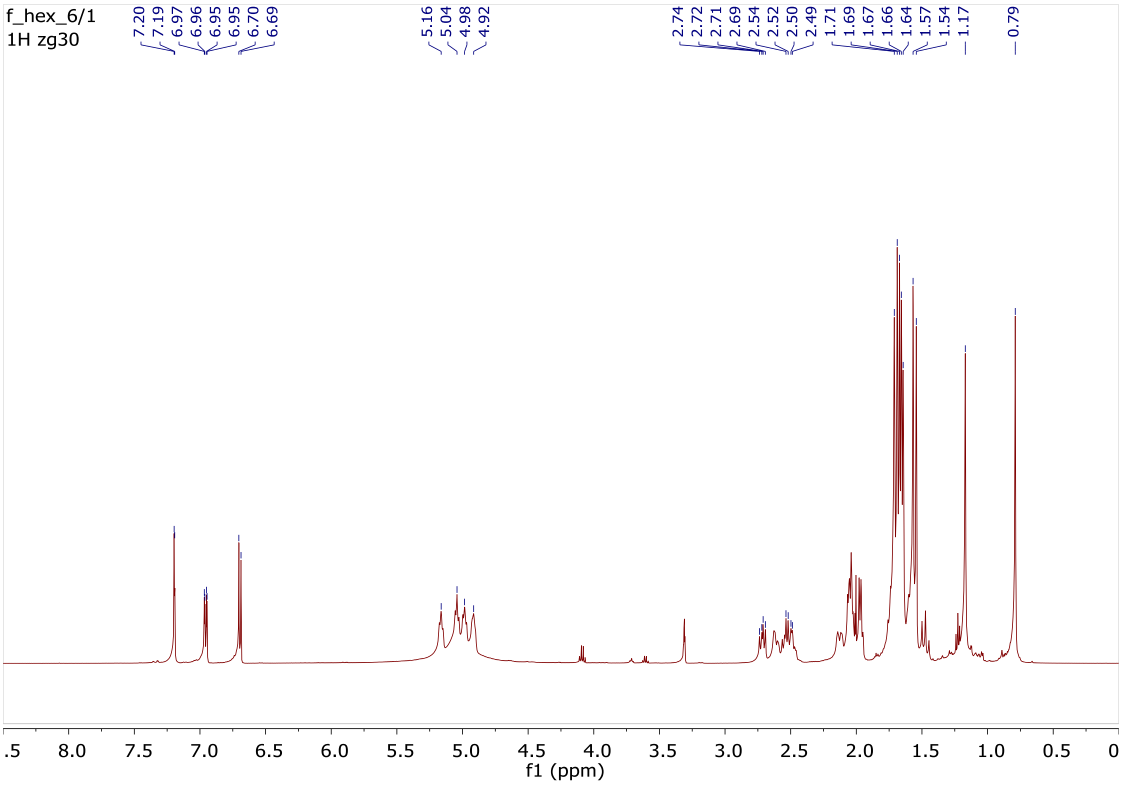

Figure 5. ^{1}H NMR spectra of oblongifolin B (**9**), 500 MHz, CD_3OD + 0.1% TFA.

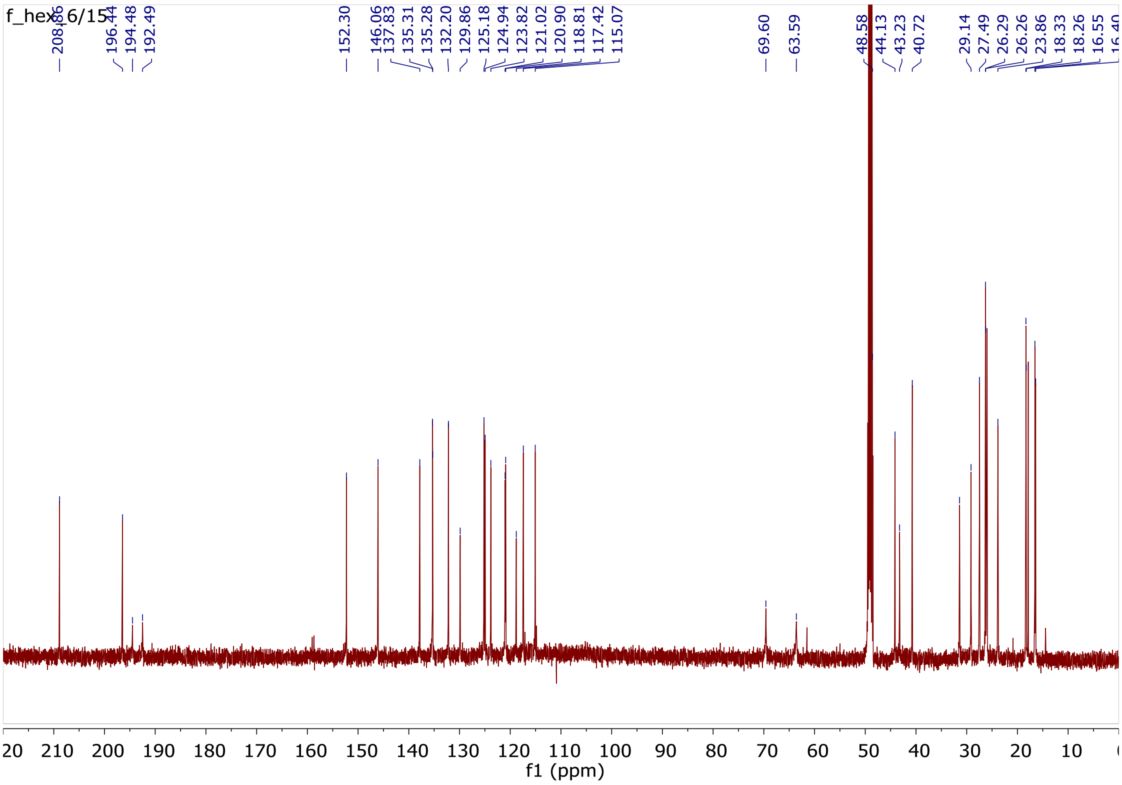

Figure 6. ^{13}C NMR spectra of oblongifolin B (**9**), 125 MHz, CD_3OD + 0.1% TFA.

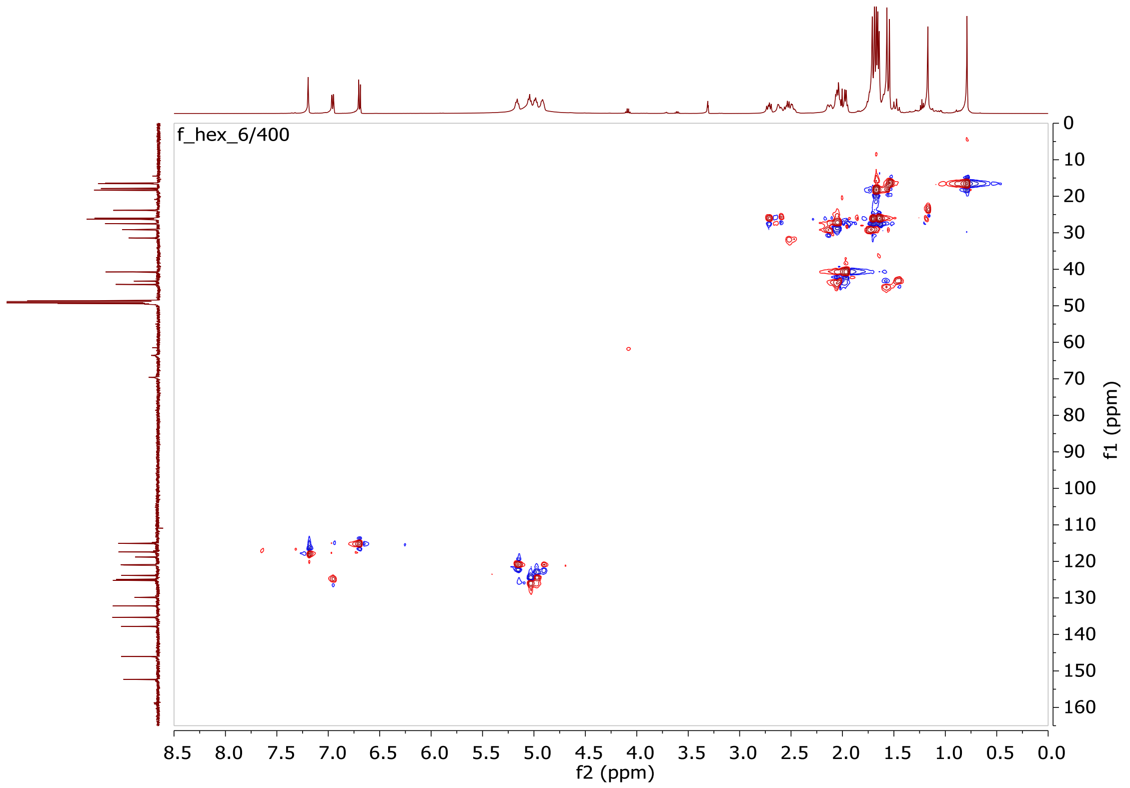

Figure 7. HSQC NMR spectra of oblongifolin B (**9**), 500 MHz, CD_3OD + 0.1% TFA.

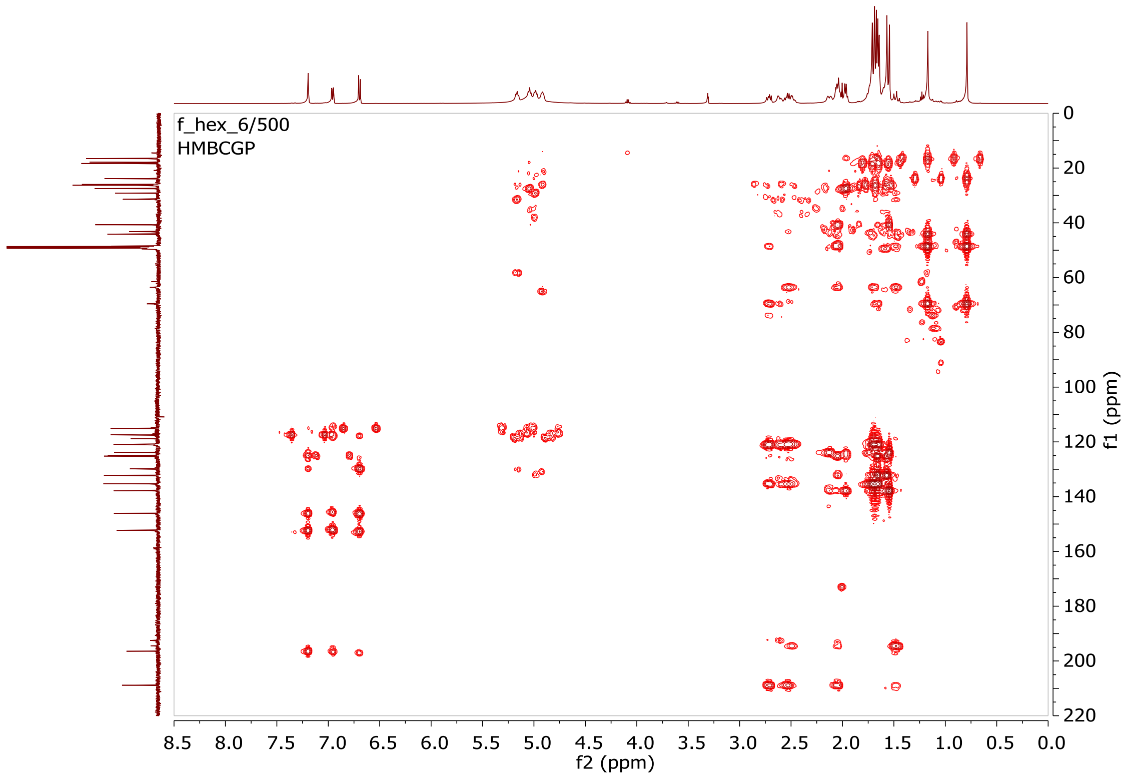

Figure 8. HMBC NMR spectra of oblongifolin B (**9**), 500 MHz, CD_3OD + 0.1% TFA.

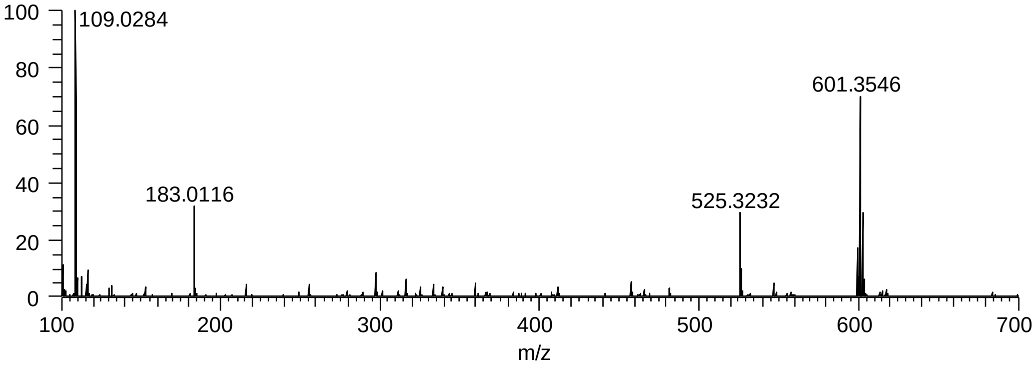

Figure 9. Negative mode HRESIMS full scan mass spectra of guttiferone E (**8**).

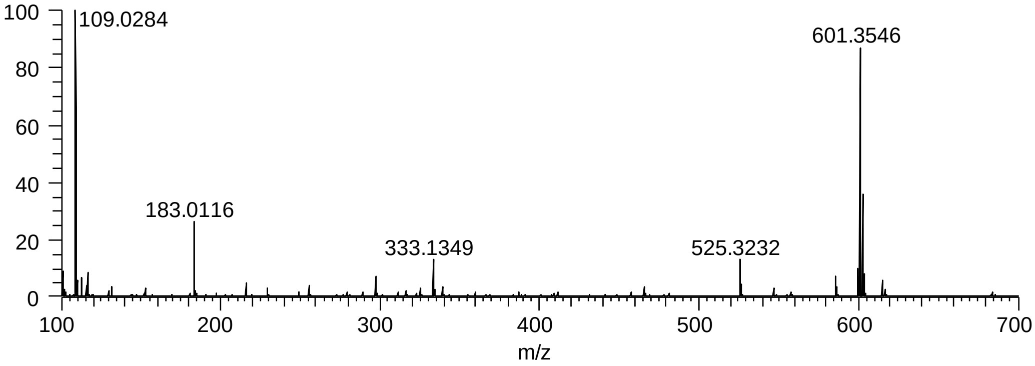

Figure 10. Negative mode HRESIMS full scan mass spectra of oblongifolin B (**9**).

Table 1. NMR data of compound **9** in comparison with the literature data of oblongifolins A and B.

	**Oblongifolin A***		**Oblongifolin B***		**Compound 9**	
Position	δ_C	δ_H, mult (*J* in Hz)	δ_C	δ_H, mult (*J* in Hz)	δ_C	δ_H, mult (*J* in Hz)
1	196.3		196.5		196.4	
2	117.9		118.9		118.8	
3	195.9		195.9		194.5	
4	68.8		69.7		69.6	
5	48.4		49		48.6	
6	47.8	1.53, m	44.2	1.60, m	44.1	1.57, m
7	40.8	eq. 2.16, m	43.3	eq. 2.05, m	43.2	eq. 2.02, m
		ax. 2.080, m		ax. 1.47, t (13.0)		ax. 1.47, t (13.1)
8	61.8		64.3		63.6	
9	209.8		209.1		208.9	
10	195.8		195.8		192.5	
11	129.5		130		129.7	
12	117.4	7.17, d (2.1)	117.4	7.20, d (2.1)	117.4	7.19, d (2.1)
13	146.2		146.1		146.1	
14	152.5		152.4		152.3	
15	115.2	6.70, d (8.3)	115.2	6.70, d (8.3)	115.1	6.70 d (8.3)
16	125.1	6.79, dd (8.3, 2.1)	125.1	6.95, dd (8.3, 2.1)	124.9	6.69 d (8.3, 2.1)
17	27	2.71, dd (9.0, 13.0)	27.4	2.71, dd (9.0, 13.0)	27.49	2.71 dd (8.6, 13.5)
		2.58, m		2.61, m		2.62, m
18	120.7	4.94, m	121.1	4.90, m	121	4.92, m
19	135.5		135.5		135.3	
20	26.3	1.71, s	26.5	1.71, s	26.3	1.71, s
21	18.3	1.67, s	18.5	1.67, s	18.3	1.67, s
22	27.4	1.01, s	16.5	0.79, s	16.4	0.79, s
23	23.3	1.24, s	24	1.17, s	23.9	1.17, s
24	30.1	2.15, m	29.2	2.13, m	29.1	2.13, m
		2.06, m		1.74, m		1.74, m
25	125.6	4.90, m	123.9	4.98, m	123.8	4.98, m
26	137.3		138		137.8	
27	16.4	1.47, s	16.6	1.54, s	16.6	1.54, s
28	40.8	1.96, m	40.8	1.96, m	40.7	1.96, m
29	32	2.54, dd (8.0, 14.0)	31.5	2.54, dd (8.0, 14.0)	31.4	2.51, dd (8.0, 14.0)
		2.47, m		2.47, m		2.47, m
30	120.7	5.16, m	120.9	5.14, m	120.9	5.16, m
31	135.7		135.4		135.3	
32	26.3	1.69, s	26.1	1.64, s	26.3	1.64, s
33	18.3	1.67, s	18.4	1.66, s	18.3	1.66, s
34	27.5	2.06, m	27.6	2.06, m	27.5	2.06, m
35	125.1	5.06, m	125.2	5.04, m	125.2	5.04, m
36	132.1		132.3		132.2	
37	26	1.65, s	26	1.65, s	26	1.64, s
38	17.8	1.56, s	18	1.58, s	17.9	1.57. s

*NMR data (CD_3OD + 0.1% TFA) published by Hamed et al., 2006 [12].
